# A Novel Protein Phosphatase 1-Dependent Spindle Checkpoint Silencing Mechanism

**DOI:** 10.1016/j.cub.2009.05.060

**Published:** 2009-07-28

**Authors:** Vincent Vanoosthuyse, Kevin G. Hardwick

**Affiliations:** 1 Wellcome Trust Centre for Cell Biology, Institute of Cell Biology, University of Edinburgh, Edinburgh EH9 3JR, UK

**Keywords:** CELLBIO

## Abstract

The spindle checkpoint is a surveillance system acting in mitosis to delay anaphase onset until all chromosomes are properly attached to the mitotic spindle [1, 2]. When the checkpoint is activated, the Mad2 and Mad3 proteins directly bind and inhibit Cdc20, which is an essential activator of an E3 ubiquitin ligase known as the anaphase-promoting complex (APC) [3]. When the checkpoint is satisfied, Cdc20-APC is activated and polyubiquitinates securin and cyclin, leading to the dissolution of sister chromatid cohesion and mitotic progression. Several protein kinases play critical roles in spindle checkpoint signaling, but the mechanism (or mechanisms) by which they inhibit mitotic progression remains unclear [4]. Furthermore, it is not known whether their activity needs to be reversed by protein phosphatases before anaphase onset can occur. Here we employ fission yeast to show that Aurora (Ark1) kinase activity is directly required to maintain spindle checkpoint arrest, even in the presence of many unattached kinetochores. Upon Ark1 inhibition, checkpoint complexes are disassembled and cyclin B is rapidly degraded. Importantly, checkpoint silencing and cyclin B degradation require the kinetochore-localized isoform of protein phosphatase 1 (PP1^Dis2^). We propose that PP1^Dis2^-mediated dephosphorylation of checkpoint components forms a novel spindle checkpoint silencing mechanism.

## Results

### Aurora Kinase Activity Is Required for the Maintenance of Spindle Checkpoint Arrest in the Absence of Microtubules

The role of Aurora kinases in checkpoint signaling remains controversial [5, 6]. Budding yeast and mammalian studies suggest that Aurora B is only required for checkpoint delays where kinetochores lack tension as a result of inappropriate attachments [7–9]. These studies argue that Aurora B has an indirect role in the spindle checkpoint. That role is to recognize inappropriately attached kinetochores, disrupt microtubule binding, and thereby produce unattached kinetochores [10] that can be recognized by the Mad/Bub proteins, leading to Cdc20 inhibition. However, two reports have argued that Aurora B activity is also required to respond to unattached kinetochores in *Xenopus* [11] and fission yeast [12]. In addition, we have previously shown that Aurora-dependent phosphorylation of budding yeast Mad3p is required for the checkpoint response to lack of tension at microtubule-bound kinetochores [6]. These findings argue that Aurora kinase has one or more direct roles to play in spindle checkpoint signaling in addition to the production of unattached kinetochores.

**Figure 1:**
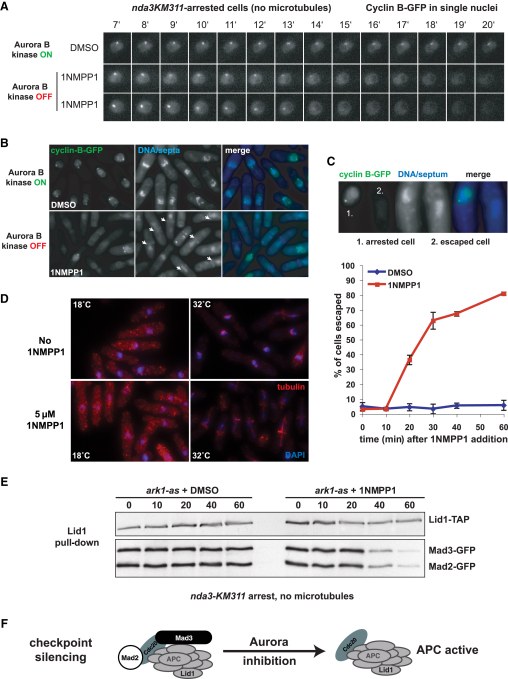
Ark1 Activity Is Required to Maintain the Mitotic Checkpoint Complex/Anaphase-Promoting Complex and Cell-Cycle Arrest (A) In the presence of the ATP analog 1NMPP1, Ark1 is the only kinase directly inhibited in *ark1-as3* cells [13]. The spindle checkpoint was activated via the cold-sensitive tubulin mutant *nda3-KM311*. After 6 hr at the restrictive temperature, 80%–90% of cells were arrested with high levels of cyclin B on spindle pole bodies (SPBs), and all kinetochores were unattached. At this point, DMSO (control) or 1NMPP1 was added to the culture to inhibit Ark1, and the levels of cyclin B were monitored by live-cell imaging. In the presence of 1NMPP1, cyclin B levels dropped rapidly, demonstrating that Ark1 activity is required to maintain spindle checkpoint arrest in response to unattached kinetochores. The time after DMSO or 1NMPP1 addition is indicated in minutes. (B) Cells were fixed 40 min after addition of 1NMPP1. Most cells escaped arrest and underwent cytokinesis (arrows). (C) Top: after addition of 1NMPP1, a few cells remain arrested with high levels of cyclin B on SPBs (cell 1), whereas most escaped the arrest and precociously degraded cyclin B (cell 2). Bottom: the number of “escaped” cells was quantified in a 60 min time course. Data are derived from four independent experiments; error bars represent standard deviation. (D) 1NMPP1 addition and Ark1 inhibition have no effect on microtubule depolymerization in the *nda3KM311* mutant. Anti-tubulin immunofluorescence confirms that at 18°C, all microtubules are depolymerized, whereas spindles are formed 15 min after release at 32°C. (E) Mitotic checkpoint complex/anaphase-promoting complex (MCC-APC) complexes are disassembled following Aurora inhibition with 1NMPP1. *Lid1-TAP ark1-as3 Mad2-GFP Mad3-GFP nda3KM311* cells were arrested in mitosis (6 hr at 18°C), and the culture was divided before DMSO or 5 μM 1NMPP1 addition. Lid1p was immunoprecipitated from samples collected every 10 min and immunoblotted for associated Mad2-GFP and Mad3-GFP. (F) Model of MCC-APC binding.

Fission yeast has only one Aurora kinase (Ark1), and this kinase is known to carry out the Aurora B role of correcting inappropriate kinetochore-microtubule attachments [13]. It was previously argued by means of *ark1* promoter shutoff and conditional *survivin* alleles that Aurora function is required to respond to unattached kinetochores [12]. Because these experiments took several hours to inactivate Ark1 kinase, we wanted to confirm the result. A conditional, ATP analog-sensitive allele of Aurora kinase (*ark1-as3*) was recently described in fission yeast [13]. With this strain, we can activate the spindle checkpoint and then inhibit Aurora kinase activity simply by adding the ATP analog (1NMPP1) to cells. Microtubule depolymerization via the cold-sensitive tubulin mutant *nda3-KM311* [14] results in a robust, spindle-checkpoint-dependent mitotic arrest, because all kinetochores are unattached [15, 16]. *nda3*-arrested cells accumulate high levels of cyclin B (Cdc13) on spindle pole bodies (SPBs) (see Figure 1). To directly test whether Aurora kinase activity is required to maintain spindle checkpoint arrest in fission yeast, we made movies of arrested *nda3ark1-as3* cells, added 1NMPP1, and followed cyclin B levels by microscopy. The SPB-enriched nuclear Cdc13-GFP signal decreased within 15 min of Aurora inhibition (Figure 1A), and many cells decondensed their chromosomes and septated (Figure 1B). We quantitated the fraction of arrested and escaped cells (Figure 1C) in a 60 min time course, during which cells were maintained at 18°C with no microtubules and unattached kinetochores (Figure 1D). In fission yeast, Mad2p and Mad3p bind to the anaphase-promoting complex (APC) upon checkpoint arrest [17], and the escape from mitotic arrest upon Ark1 inhibition correlated well with the loss of Mad3p- and Mad2p-APC binding (Figures 1E and 1F). Note that in this assay, the *nda3* cells were maintained at 18°C throughout, spindles did not form, and all kinetochores remained unattached (Figure 1D). Thus, we directly assayed the ability of cells to degrade cyclin B after Ark1 inhibition independently of its role in kinetochore-microtubule attachment and error correction processes. A similar result was obtained when an experiment was performed at 32°C in the presence of the microtubule-depolymerizing drug carbendazim, ruling out the possibility that cyclin B degradation upon Ark1 inhibition is a side effect of growing cells at 18°C (see Figure S1 available online). We conclude that Ark1 activity is required to maintain spindle checkpoint arrest, even in the presence of many unattached kinetochores.

### PP1 Phosphatase Is Necessary for Checkpoint Silencing

Although checkpoint activation is well characterized, far less is understood about how the spindle checkpoint is silenced when all sister chromatids are attached to spindle microtubules in an appropriate fashion. Checkpoint silencing is an important final step prior to anaphase onset and needs to be both efficient and well coordinated to ensure a synchronous anaphase. Two major silencing mechanisms have been proposed: (1) dynein- and microtubule-dependent “stripping” of Mad2 from kinetochores [18], and (2) disruption of Mad2-Cdc20 complexes by either p31^comet^ [19, 20] or Cdc20 ubiquitylation [21].

**Figure 2:**
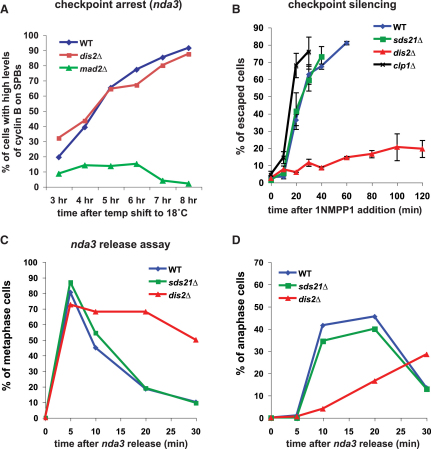
PP1^Dis2^ Phosphatase Is Required for Checkpoint Silencing Following Ark1 Inhibition, and *dis2* Mutants Have a Delay in Anaphase Onset upon Spindle Reassembly (A) When shifted to their restrictive temperature of 18°C, *nda3-KM311* cells depolymerize microtubules and activate the spindle checkpoint with unattached kinetochores. Over time, *nda3-KM311* and *nda3KM311 dis2*Δ cells accumulate in early mitosis with high levels of cyclin B (Cdc13-GFP) in the nucleus and on SPBs, showing that, unlike Mad2p, Dis2p is not required for checkpoint arrest. (B) The silencing assay was used to test whether phosphatases played a role in checkpoint silencing. Strikingly, when 1NMPP1 was added to the cultures, APC activation and cyclin B degradation were negligible in the absence of PP1^Dis2^ (*dis2*Δ), suggesting that PP1^Dis2^ is critical for checkpoint silencing upon Ark1 inhibition. This is a specific defect, because *sds21*Δ and *clp1*Δ mutants behaved like wild-type (as did PP2A^Par1^ mutants; data not shown). For simplicity, the DMSO controls are not shown here. Data are derived from a minimum of three experiments; error bars represent standard deviation. (C and D) *nda3KM311*-arrested cells (6 hr at 18°C) were allowed to reform their spindle at the permissive temperature of 32°C (see Experimental Procedures). Progression through mitosis was then followed at regular intervals using spindle length as a marker, scoring the percentage of metaphase (C) and anaphase (D) cells. Upon recovery from *nda3KM311* arrest, lack of Dis2p induced ∼20 min metaphase delay, whereas lack of Sds21p had no effect on the kinetics of anaphase onset.

It has proven difficult to isolate mutants and devise assays that specifically address the mechanism (or mechanisms) of checkpoint silencing. This is due to the intimate link between biorientation and checkpoint signaling: an apparent failure to silence the checkpoint could be caused by prolonged checkpoint activation if defective kinetochore-microtubule interactions persist. The *nda3ark1-as3* “silencing” assay described above (Figure 1) is independent of chromosome attachment and biorientation and is thus specific for checkpoint silencing and APC activation. We used this assay to screen fission yeast mutants and thereby identify factors that act downstream of Ark1 during checkpoint silencing. Silencing mutants should fail to degrade cyclin B upon *ark1-as3* inhibition. In particular, we tested whether protein phosphatase activity was necessary to silence the checkpoint. PP1 phosphatase has been argued to oppose Aurora kinase in a number of systems [22–24]. Fission yeast expresses two PP1 phosphatases, PP1^Dis2^ and PP1^Sds21^ [25], and PP1^Dis2^ has been localized to kinetochores ([26] and data not shown). To analyze the role of PP1, we constructed *dis2*Δ*nda3* and *sds21*Δ*nda3* strains. These phosphatase mutants undergo spindle checkpoint arrest efficiently (Figure 2A). Strikingly, addition of the Ark1 inhibitor 1NMPP1 to *dis2*Δ*nda3ark1-as3*-arrested cells failed to result in cyclin B degradation, even after 2 hr (Figure 2B). This silencing defect was highly specific: lack of the other PP1 phosphatase (PP1^Sds21^) or of two other centromeric phosphatases (PP2A^Par1^ and the Cdc14 homolog Flp1p/Clp1p) did not delay cyclin B degradation upon Aurora inhibition (Figure 2B and data not shown). This assay provides direct evidence for a role for PP1^Dis2^ in spindle checkpoint silencing.

To look for a silencing defect under more physiological conditions, we analyzed the recovery of *dis2*Δ cells from an *nda3* block, where checkpoint silencing takes place after spindle reassembly and chromosome attachment. When *nda3* cells were shifted back to their permissive temperature (32°C), they efficiently assembled a spindle and typically entered anaphase within 10–20 min (Figure 1D). We compared *nda3* and *dis2*Δ*nda3* cell populations and observed a clear delay in anaphase onset following spindle reassembly in *dis2*Δ*nda3* (Figures 2C and 2D). Again, this phenotype was specific to the loss of PP1^Dis2^, because *sds21*Δ*nda3* mutants released with wild-type kinetics (Figures 2C and 2D). This demonstrates that, in addition to inhibition of Ark1 kinase activity, the action of kinetochore-localized PP1^Dis2^ is needed for efficient spindle checkpoint silencing.

### Discussion

This study has two important findings highlighting the critical balance between Aurora kinase and PP1 activities in the regulation of anaphase onset:

(1) Ark1 activity is required to maintain a spindle checkpoint arrest, even in the presence of many unattached kinetochores (see Supplemental Discussion on Aurora kinases and checkpoint signaling). This suggests that Ark1 has important Mad/Bub checkpoint substrates, which is consistent with our previously published budding yeast work [6].
(2) We describe a novel role for kinetochore-localized protein phosphatase 1, PP1^Dis2^, in spindle checkpoint silencing. We propose that PP1^Dis2^ has one or more Mad/Bub protein substrates and do not rule out Cdc20-APC as another important target that needs to be dephosphorylated before the checkpoint is silenced and the APC activated. In a separate study, we have shown that APC activation upon Ark1 inhibition requires the Bub3 checkpoint component (unpublished data). Mad3/BubR1, Bub3, and Bub1 are all found at kinetochores later in mitosis than Mad1 and Mad2, which are no longer recruited to attached kinetochores. Therefore, we believe that the Bub3p binding partners (Bub1p and Mad3p) are both strong candidates for important Aurora and PP1^Dis2^ substrates. To date, we have mapped >70 phosphorylation sites on these two proteins, and their functional dissection is a major goal of ongoing studies.

**Figure 3:**
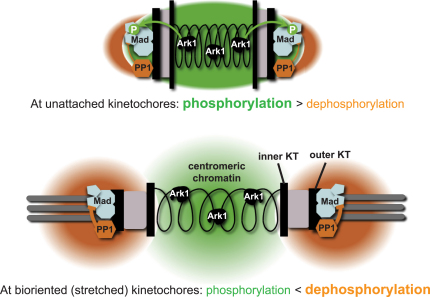
Silencing Model Top: at unattached kinetochores, centromeric chromatin is not under tension, and centromere-localized Ark1 kinase (the green cloud of activity) is in close proximity to kinetochore-localized checkpoint components and can phosphorylate them efficiently, leading to effective APC inhibition. Bottom: stable attachments are formed after biorientation and lead to inter- and intrakinetochore stretching. This displaces Ark1 from kinetochore substrates and reduces its ability to phosphorylate checkpoint components, which can in turn be dephosphorylated by PP1^Dis2^ (orange clouds of activity), leading to checkpoint silencing.

We propose the following simple model to explain how PP1 activity toward the kinetochore is regulated by kinetochore attachment to microtubules (Figure 3). Upon chromosome biorientation, inter- and intrakinetochore stretching spatially separates Aurora B kinase from outer kinetochore substrates, such as Ndc80 and MCAK, and thereby stabilizes kinetochore-microtubule attachments [27–30]. In the same way, this stretching could separate Aurora B from relevant checkpoint targets, such as BubR1/Mad3 [6], whose modification is important for spindle checkpoint arrest (Figure 3). Physical separation of Aurora B from kinetochore substrates contributes to the stabilization of kinetochore-microtubule attachments. In turn, this stimulates the dynein- and microtubule-dependent kinetochore stripping of spindle checkpoint components, whereby Mad2 is transported from kinetochores to spindle poles and then released [18, 31, 32]. This pathway has been proposed to contribute to spindle checkpoint silencing, and it is regulated by the kinetochore-localized PP1γ isoform in mammalian cells [33]. However, it is unclear whether stripping of Mad2 has a direct consequence on APC activation, and anaphase onset can occur without kinetochore stripping of Mad2 and BubR1 [34]. Note that the silencing in our *nda3ark1-as3* assay occurs in a microtubule-independent manner (Figure 1) and can therefore be independent of kinetochore-stripping of checkpoint components.

Several other silencing mechanisms have been proposed. p31^comet^ can disrupt Mad2-Cdc20 complexes and thereby induce anaphase onset [19, 20, 35]. However there are no known yeast homologs of p31^comet^, and it is not yet clear how p31^comet^ function is regulated by kinetochore attachment. Cdc20 ubiquitylation might lead to disruption of Mad2/BubR1 binding and thereby relieve checkpoint inhibition [21]. However, a nonubiquitylatable Cdc20 mutant where all lysine residues had been replaced with arginine was recently shown to have little difficulty in exiting spindle checkpoint arrests [36]. In vertebrates, CENP-E-BubR1 interactions have been proposed to silence the checkpoint upon chromosome attachment [37], but yeast and *C. elegans* lack a CENP-E homolog, and their Mad3 lacks a C-terminal kinase domain. Finally, in budding yeast it has been argued that Mps1 kinase is degraded to prevent checkpoint signaling in anaphase [38], but this has yet to be confirmed in other systems. Although there could be several ways to turn off the spindle checkpoint, we propose that PP1 plays an important role in several of these silencing mechanisms. Our findings on the role of PP1 in spindle checkpoint silencing are strongly supported by a study of the budding yeast PP1 homolog Glc7 appearing in this issue of *Current Biology* [39].

### Conclusions

We have developed a novel assay completely independent of kinetochore-microtubule attachment to dissect spindle checkpoint silencing mechanisms acting downstream of Aurora kinase inhibition. This has enabled us to identify PP1^Dis2^ as a key silencing component. A more in-depth analysis of the silencing mechanism (or mechanisms) and how it is coordinated with biorientation will be important for a full understanding of mitotic regulation and may also be relevant to human disease. Indeed, the mitotic exit mechanisms utilized by cells, particularly during clinical treatment of cancers with antimicrotubule drugs such as taxanes, may in part determine their fate and thereby the effectiveness of therapies [40, 41].

## Experimental Procedures

### Yeast Strains

A complete list of all of the strains used in this study is given in Table S1.

### Microscopy

Microscopy was performed as described previously [16] with an Intelligent Imaging Innovations (3i) Marianas microscope, CoolSnap CCD, and SlideBook software (3i). For live-cell imaging in Figure 1A, mid-log *ark1-as3 cdc13-GFP nda3-KM311* cells were first arrested in early mitosis in liquid cultures by shifting the temperature to 18°C for 6 hr. Precooled dimethyl sulfoxide (DMSO) or 5 μM 1NMPP1 was added to the culture, and cells were immediately mounted on precooled (18°C) slides and coverslips and imaged on a precooled (18°C) microscope stage. Images were acquired every minute. At least ten movies were acquired for each condition.

### Checkpoint Silencing Assay

Mid-log *ark1-as3 cdc13-GFP nda3-KM311* cells were arrested in early mitosis in liquid cultures by shifting to 18°C for 6 hr. The culture was divided in two, and DMSO or 5 μM 1NMPP1 was added. Cells (300 μl) were fixed every 10 min by mixing with 1 ml 100% methanol and mounted immediately in medium containing DAPI (to label DNA) and calcofluor (to label cell walls and septa). One hundred cells for each treatment condition (DMSO or 1NMPP1) were counted immediately under the microscope before the next time point.

### Mitotic Checkpoint Complex/Anaphase-Promoting Complex Interaction

Cells expressing TAP-tagged Lid1 (Apc4) and Mad2p and Mad3p tagged with GFP from their endogenous loci were presynchronized either in G2 via the *cdc25-22* mutation (Figure 3) or in early mitosis via the *nda3-KM311* mutation (Figure 1). Proteins were extracted in lysis buffer (50 mM HEPES [pH 7.6], 75 mM KCl, 1 mM MgCl_2_, 1 mM EGTA, 0.1% Triton, 1 mM sodium vanadate, microcystin, leupeptin, pepstatin, chymostatin, and Pefabloc) from ∼2 × 10^8^ cells as described previously [42]. Extracts were incubated for 30 min with IgG-coupled Dynabeads, which bind to Lid1-TAP. The immunoprecipitated complexes were washed three times with lysis buffer and once with phosphate-buffered saline containing 0.02% Tween 20. Immunoprecipitated complexes were analyzed by immunoblot with a sheep anti-GFP antibody.

### nda3-KM311 Release Assay

Mid-log *nda3-KM311* cells were arrested in early mitosis in liquid cultures by shifting the temperature to 18°C for 6 hr. Cells were then filtered on a Durapore 0.45 μm HV filter (Millipore) and released from the filter into prewarmed medium (32°C) by shaking. At each time point following release at 32°C, cells (2 ml) were fixed by mixing with 20 ml 100% methanol precooled to −80°C. Cells were then processed for immunofluorescence with an anti-tubulin antibody (TAT1, a kind gift of K. Gull, University of Oxford).

## Supplemental Data

Document S1. Supplemental Discussion, One Table, and One Figure
