# Supplementary material for: A Novel Protein Phosphatase 1-Dependent Spindle Checkpoint Silencing Mechanism
Source: Curr Biol. 2009 Jul 28;19(14-3):1176–81. doi: 10.1016/j.cub.2009.05.060 (PMC2791888; doi:10.1016/j.cub.2009.05.060)
Supplement: Document S1. Supplemental Discussion, One Table, and One Figure [file mmc1.pdf]

Current Biology, Volume *19*

Supplemental Data

A Novel Protein Phosphatase 1-Dependent Spindle Checkpoint Silencing Mechanism

Vincent Vanoosthuyse and Kevin G. Hardwick

Supplemental Discussion

Aurora Kinase and Checkpoint Signaling

Budding yeast Aurora (Ipl1) kinase activates the spindle checkpoint by creating unattached kinetochores [1, 2]. This response, to inappropriately attached kinetochores, is central to the error-correction process and is widely conserved [3, 4]. Our data shows that Ark1 activity is required to maintain a spindle checkpoint arrest even in the presence of unattached kinetochores (Figure 1), demonstrating that it must also have direct checkpoint signaling functions to maintain effective inhibition of Cdc20-APC. These observations are consistent with earlier fission yeast [5] and *Xenopus* findings [6]. In the latter it was shown, in both *Xenopus* extracts and XTC cells, that Aurora B was required for maintenance of the spindle checkpoint arrest in cells with disrupted spindles.

Why is Aurora B activity not required for the response to unattached kinetochores in budding yeast or tissue culture cells [3, 4, 7]? All the studies that came to this conclusion rely on conditional alleles (*ipl1-ts*), RNAi or small molecule inhibition of Aurora B activity, and thus there is no guarantee that Aurora kinase activity was completely inhibited. In addition, we note that whilst ZM447439 treatment had little effect in the short term on vertebrate cells arrested with nocodazole, after longer periods the checkpoint arrest was profoundly compromised [3]. Perhaps residual Aurora B activity was sufficient for checkpoint signaling in these experiments where many kinetochores were unattached? Alternatively, two recent reports have proposed that Aurora A kinase could have a role in vertebrate spindle checkpoint signaling [8, 9], so it could be that Ark1 functions are carried out by distinct Aurora kinases in vertebrates. Note, there is no evidence that Ark1 has Aurora A-like spindle assembly functions in *S. pombe*. Perhaps fission yeast is simply different, with a more significant checkpoint role for Aurora kinase than other systems, but the *Xenopus* data argues against this. Further work will be necessary to clarify this issue.

We propose that Aurora kinase contributes to spindle checkpoint signaling in two ways: (A) indirectly, by destabilising kinetochore-microtubule attachments that fail to produce tension [2], and (B) by directly phosphorylating checkpoint components such as Mad3p [10] and thereby enhancing the “wait-anaphase” signal. We propose that certain Mad/Bub modifications need to be removed, by $PP1^{Dis2}$, before the APC can be activated (see Figure 3).

Supplemental References

1. Tanaka, T.U., Rachidi, N., Janke, C., Pereira, G., Galova, M., Schiebel, E., Stark, M.J., and Nasmyth, K. (2002). Evidence that the Ipl1-Sli15 (Aurora kinase-INCENP) complex promotes chromosome bi-orientation by altering kinetochore-spindle pole connections. Cell *108*, 317-329.
2. Pinsky, B.A., Kung, C., Shokat, K.M., and Biggins, S. (2006). The Ipl1-Aurora protein kinase activates the spindle checkpoint by creating unattached kinetochores. Nat Cell Biol *8*, 78-83.
3. Ditchfield, C., Johnson, V.L., Tighe, A., Ellston, R., Haworth, C., Johnson, T., Mortlock, A., Keen, N., and Taylor, S.S. (2003). Aurora B couples chromosome alignment with anaphase by targeting BubR1, Mad2, and Cenp-E to kinetochores. J Cell Biol *161*, 267-280.
4. Hauf, S., Cole, R.W., LaTerra, S., Zimmer, C., Schnapp, G., Walter, R., Heckel, A., van Meel, J., Rieder, C.L., and Peters, J.M. (2003). The small molecule Hesperadin reveals a role for Aurora B in correcting kinetochore-microtubule attachment and in maintaining the spindle assembly checkpoint. J Cell Biol *161*, 281-294.
5. Petersen, J., and Hagan, I.M. (2003). S. pombe aurora kinase/survivin is required for chromosome condensation and the spindle checkpoint attachment response. Curr Biol *13*, 590-597.
6. Kallio, M.J., McCleland, M.L., Stukenberg, P.T., and Gorbsky, G.J. (2002). Inhibition of aurora B kinase blocks chromosome segregation, overrides the spindle checkpoint, and perturbs microtubule dynamics in mitosis. Curr Biol *12*, 900-905.
7. Biggins, S., and Murray, A.W. (2001). The budding yeast protein kinase Ipl1/Aurora allows the absence of tension to activate the spindle checkpoint. Genes Dev *15*, 3118-3129.
8. Stolz, A., Vogel, C., Schneider, V., Ertych, N., Kienitz, A., Yu, H., and Bastians, H. (2009). Pharmacologic abrogation of the mitotic spindle checkpoint by an indolocarbazole discovered by cellular screening efficiently kills cancer cells. Cancer Res *69*, 3874-3883.
9. Wysong, D.R., Chakravarty, A., Hoar, K., and Ecsedy, J.A. (2009). The inhibition of Aurora A abrogates the mitotic delay induced by microtubule perturbing agents. Cell Cycle *8*, 876-888.
10. King, E.M., Rachidi, N., Morrice, N., Hardwick, K.G., and Stark, M.J. (2007). Ipl1p-dependent phosphorylation of Mad3p is required for the spindle checkpoint response to lack of tension at kinetochores. Genes Dev *21*, 1163-1168.

Table S1. Fission Yeast Strains Used in This Study

yVV1381	h+ ark1-as3::hyg+ cdc13-GFP::LEU2 nda3-KM311
yVV1437	Lid1-TAP::G418 ark1-as3::hygB+ Mad2-GFP::his3+ Mad3-GFP::his3+ nda3-KM311
yVV1388	ark1-as3::hygB+ cdc13-GFP::LEU2 dis2Δ::ura4+ nda3-KM311
yVV1420	ark1-as3::hygB+ cdc13-GFP::LEU2 par1Δ::G418 nda3-KM311
yVV1435	ark1-as3::hygB+ cdc13-GFP::LEU2 sds21Δ::leu2+ nda3-KM311
yVV1439	ark1-as3::hygB+ cdc13-GFP::LEU2 clp1Δ::ura4+ nda3-KM311
yVV429	cdc13-GFP::LEU2 cut12-CFP::G418 nda3-KM311
yVV1369	cdc13-GFP::LEU2 mad2Δ::ura4+ cut12-CFP::G418 nda3-KM311
yVV54	h+ nda3-KM311
yVV1215	h+ dis2Δ::ura4+ nda3-KM311

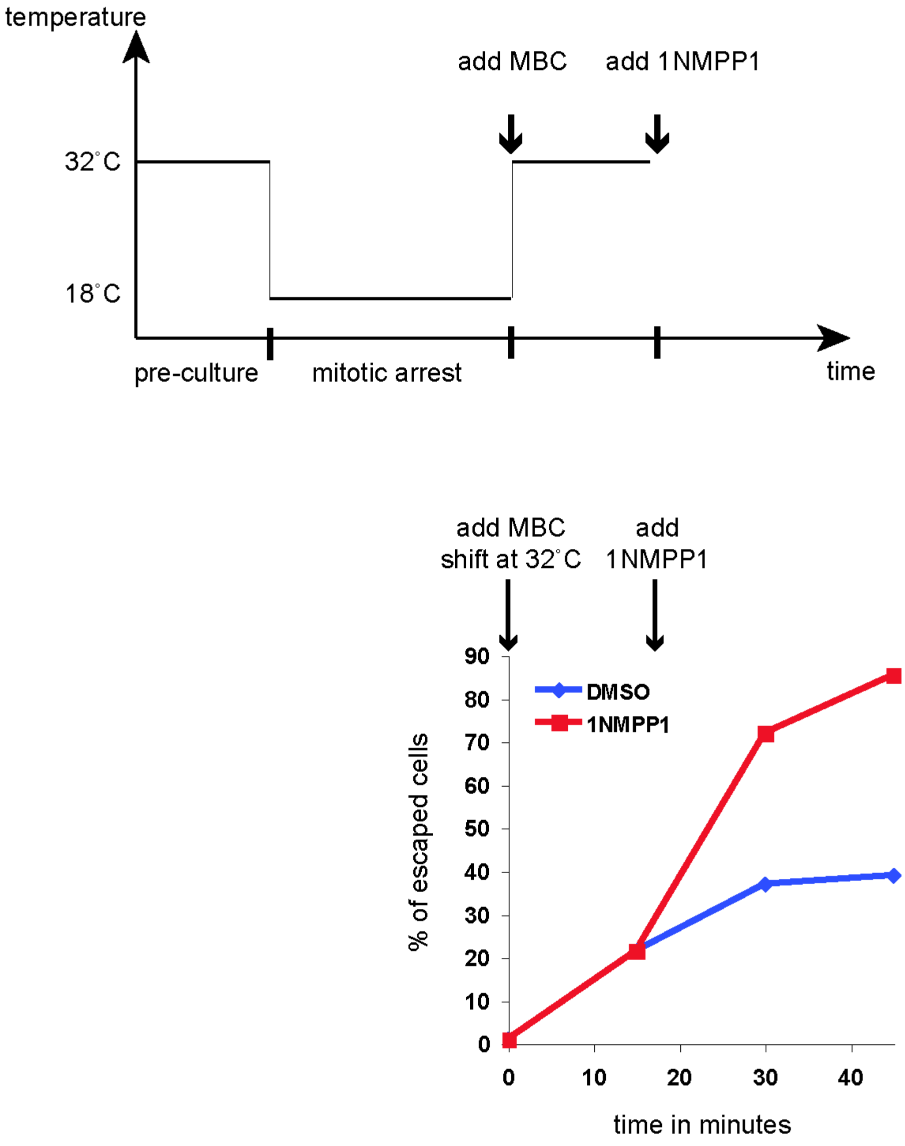

Figure S1. Ark1 Inhibition Also Leads to APC Activation at 32˚C
To rule out that cyclin B degradation upon Ark1 inhibition is a side-effect of growing cells at 18˚C, cells expressing the Shokat allele of Ark1 (*ark1-as3*) were first arrested at 18˚C for 6 hours, then released at 32˚C in the presence of 50µg/mL of the microtubule-depolymerising drug carbendazim (CBZ) for 15 minutes. The culture was then split in two and the Ark1 inhibitor 1NMPP1 was added to one of the two cultures. The percentage of "escaped" cells was then counted as in Figure 1.
